# Effects of word list length during episodic memory encoding observation by the event-related potential and time-frequency

**DOI:** 10.3389/fnhum.2025.1542289

**Published:** 2025-04-22

**Authors:** Viktors Veliks, Aleksandrs Kolesovs, Juris Porozovs, Dmitrijs Igonins

**Affiliations:** University of Latvia, Riga, Latvia

**Keywords:** memory, electroencephalography, ERP, TF, word lists, information encoding

## Abstract

The present study explored the effects of word list length during the encoding of visual verbal stimuli. The participants received Latvian nouns in lists of different lengths: short (up to 29), medium (30–59), and long (60–160). During the presentation of visual stimuli, the 19–channel EEG was recorded with a sample rate of 512 Hz and cut-off frequencies of 0.1–50 Hz. The memory encoding process was analyzed with the event-related potential (ERP) and time-frequency (TF) methods for selected regions of interest (ROI) electrodes F3, F7, C3, P3, T3, and T5 in the 10–20 system corresponding to language processing brain areas. We compared ERP and TF data regarding the list length in the −100 ms to 700 ms time window. ROI electrodes T3, T5, and P3 indicated significantly different involvement of language processing areas under different list lengths by ERP observation. More lateralized regions (F7, T3) provided evidence for more pronounced differences in the encoding process than less lateralized regions (F3, C3). The analysis of TF revealed differences in theta, alpha, and beta wave bands in the F3 and P3 channels. Medium lists demonstrated higher differences from short and long lists, indicating a nonlinear trend in the involvement of language-processing regions.

## 1 Introduction

The growing flow of information increases cognitive load, leading to stress. It negatively affects professionals, including medical workers (Lafraxo et al., 2021) or cabin crew (Kim et al., 2022). Increasing stress can impair cognitive functioning in general and memory in particular (Schwabe and Wolf, 2012), which results in attention loss and errors with negative consequences for people or industries (Kim et al., 2022; Pooladvand and Hasanzadeh, 2022). Mechanisms of impaired functioning involve suppression of working memory – a component of memory responsible for storing and manipulating information during the performance and ensuring complex interaction with long-term memory (Baddeley, 2012). A recent study (Park et al., 2023) demonstrated that additional working memory load results in difficulties in impulse control and reduced learning. The significance of memory for metacognition (e.g., Drigas and Mitsea, 2021) also emphasizes the need for further investigation of human functioning under increasing cognitive load conditions.

Encoding is the crucial step in memory stages, which ensures the transformation of events and thoughts into neural representations for their maintenance and following recall or recognition (Craik and Rose, 2012). Neurocognitive theories have been developed to explain information encoding in long-term memory. According to the hemispheric encoding/retrieval asymmetry (HERA) model (e.g., Tulving et al., 1994), the left prefrontal cortex (PFC) is more involved in the process of encoding than in the retrieval of information. This asymmetry can be observed in the encoding and retrieval of short- and long-term episodic memory with different types of materials (Blanchet et al., 2001) and is the most pronounced for verbal stimuli (Habib et al., 2003).

Jeneson and Squire (2012) emphasized in a review that the involvement of brain regions in encoding is associated with cognitive load. When the amount of information exceeds the capacity of working memory, temporal and parietal lobes are more actively involved, together with the prefrontal region primarily associated with this type of memory. As Chai et al. (2018) indicated, this process reflects the activation of the attention controller, phonological loop, and episodic buffer. Long-term memory is encoded and maintained by neurons and glial cells distributed across multiple regions. These neuron ensembles are reactivated during the retrieval of information. Differences in connections of dendrites in neuronal ensembles form individual variability in long-term memory (Karaca et al., 2021). It is emphasized that memory retrieval occurs in the hippocampal assemblies and other brain regions during sleep (Brodt et al., 2023). Other authors emphasize the possibility of strengthening memory by forming cognitive links between new information and existing knowledge (e.g., Siagian et al., 2023). Therefore, encoding information into long-term memory depends on various factors, including different neural mechanisms, cognitive strategies, and contextual factors.

In parallel with research on memory, continuous discussions on language neurobiology (Fedorenko and Blank, 2020; Tremblay and Dick, 2016) directly support the involvement of frontal, temporal, and parietal regions in verbal information processing. Studies on brain asymmetry (Morillon et al., 2010) confirm the domination of left-hemispheric structures in speech and language processing, which overlap with neural correlates of encoding during short-memory tasks involving the inferior frontal, inferior parietal, and superior temporal cortex (Wiesman et al., 2021).

Previous studies predominantly focused on maintenance and recognition processes and their association with the volume of information under consideration, including the length of presented lists of words (e.g., Kinnell and Dennis, 2011). Consequently, encoding processes have received less attention in neurocognitive studies related to the effects of working memory load (Bashivan et al., 2014). Studies on long-term episodic memory also focus more on recognition processes than on encoding, which is typically discussed in the context of subsequent memory effects (e.g., Kahana, 2012). Serruya et al. (2014) pointed out the importance of recording neural activity at the encoding stage to establish differences in the neurocognitive processing of initial and subsequent elements of memorized lists.

It should be noted that studies typically used list lengths of up to 100 items (Cary and Reder, 2003; Dennis and Humphreys, 2001). This number of items included significant points for studying the extension of working memory (Ericsson and Kintsch, 1995). Simultaneously, there is a need to assess memory processes in larger sets of elements associated with other vitally significant tasks. For example, maintaining connections between individuals and groups within a social network requires addressing sets of 100 to 200 individuals (Hill and Dunbar, 2003). These set sizes are associated with limitations of human brain neural structures (e.g., Whitehead, 2010), including limitations of memory functioning and using heuristics to overcome the limit (e.g., Alessandretti et al., 2018).

Therefore, studies on memory processes under a growing information load can add to the body of knowledge. As various authors pointed out (Bashivan et al., 2014; Wiesman et al., 2021), the effects of memory load on neurocognitive encoding processes have received less attention and remain to be further investigated. Extending the number of visual stimuli aligns with current tendencies in online information flow. Non-verbal stimuli are used in research on relatively large set sizes (e.g., Torres-Trejo and Cansino, 2016). The encoding of large sets of verbal stimuli was the focus of the current study. It aimed to reveal the effects of list length on left-hemispheric brain activity while encoding words.

The study included a set of regions of interest (ROI) having high significance in encoding visual representations of words (Weiss and Rappelsberger, 2000): F3, F7, C3, P3, T3, and T5. These ROI electrodes represented significant regions of cortical activity during encoding short-term memory (Wiesman et al., 2021) and verbal information processing (Morillon et al., 2010) associated with Broca and Wernicke’s language processing areas (Tremblay and Dick, 2016). Simultaneously, they were electrophysiological correlates of working memory, including attention control, episodic buffer, and phonological loop areas (Chai et al., 2018).

In addition to differences in event-related potential, the memory load was expected to modulate brain oscillations. Theta band oscillations were expected to be closely associated with memory encoding (Gruber et al., 2013; Klimesch et al., 2005; Meltzer et al., 2007; Osipova et al., 2006). Alpha and beta bands (Griffiths et al., 2021; Herrmann et al., 2004; Meltzer et al., 2007) were also expected to be involved in memory encoding processes. Furthermore, there is evidence for more pronounced semantic processing in the theta frequency band and phonological and orthographic processing in the upper alpha band (Weber et al., 2013), which may reflect participants’ strategies during encoding lists of different lengths.

## 2 Methods

### 2.1 Participants

The study included data from 14 female volunteer students from the Riga Teacher Training and Educational Management Academy pedagogy program aged 38 ± 8 years. All participants were right-handed native Latvian speakers without vision or cognitive difficulties. The higher efficacy of females on verbal memory tasks (e.g., Wong et al., 2020) and the structure of the teachers’ population in Latvia, with over 80% females, determined forming a sample homogeneous regarding participants’ gender. The research was conducted in accordance with the national legislation of the Republic of Latvia and the Declaration of Helsinki. In the frame of scientific cooperation, the current study used anonymized data from a previous project. For the exploration of effects, G*Power 3.1 (Faul et al., 2009) calculation indicated a minimum a-priori sample size of 12 participants for a medium effect size, an alpha level of 0.05, and a statistical power of 0.80.

### 2.2 Procedure

The study followed a within-subjects design with a positionally balanced presentation of medium (M) and short (S) word lists within the first session and an additional exploratory session involving the long (L) list of words. Therefore, each participant completed five memory tasks following a sequence of M-S-S-M-L. List lengths were balanced to the number of words among participants within the first session.

Word stimuli in the present study were nouns selected from the first 1,500 most common words in the Latvian spoken language. Five tasks with different lengths were prepared for each participant and divided into two separate sessions, balancing the total number of encoded words. Words were manually selected and formed following tasks: two tasks with a list length of 1–29 words (short), two tasks with a list length of 30–59 words (medium), and one task with a list length of 60–160 words (long).

Word characteristics were balanced by a random selection of words from the list of the most frequent words. Each participant had their own combination of words in possible lists. Concrete and abstract nouns of different lengths (number of letters from 3 to 14, mean±SD was 6.58 ± 1.89) were used in this study. Compound nouns were not included. Word examples: “Kartupelis” (Potato), “Lācis” (Bear), “Zvaigzne” (Star), “Doma” (Thought), “Darbība” (Action), “Gadījums” (Chance).

Participants’ attentional control methods were not used during the encoding task, given evidence that neural representations of high-frequency words are automatically activated when words are presented, even when they are not the primary focus of attention (Alexandrov et al., 2011; Shtyrov and Pulvermüller, 2007).

The experiment was performed in a quiet room. During the recording, participants assumed a comfortable position in a chair. During the experiment, the light was turned off.

Psychtoolbox-3 software^1^ was used for stimulus presentation. Black-colored words (Arial fonts and size of 50 pixels) were presented on a white screen. Stimulus words were presented on a 17” LCD resolution 1200×1024 monitor, and participants were seated approximately 100 cm away from it.

**Figure 1:**
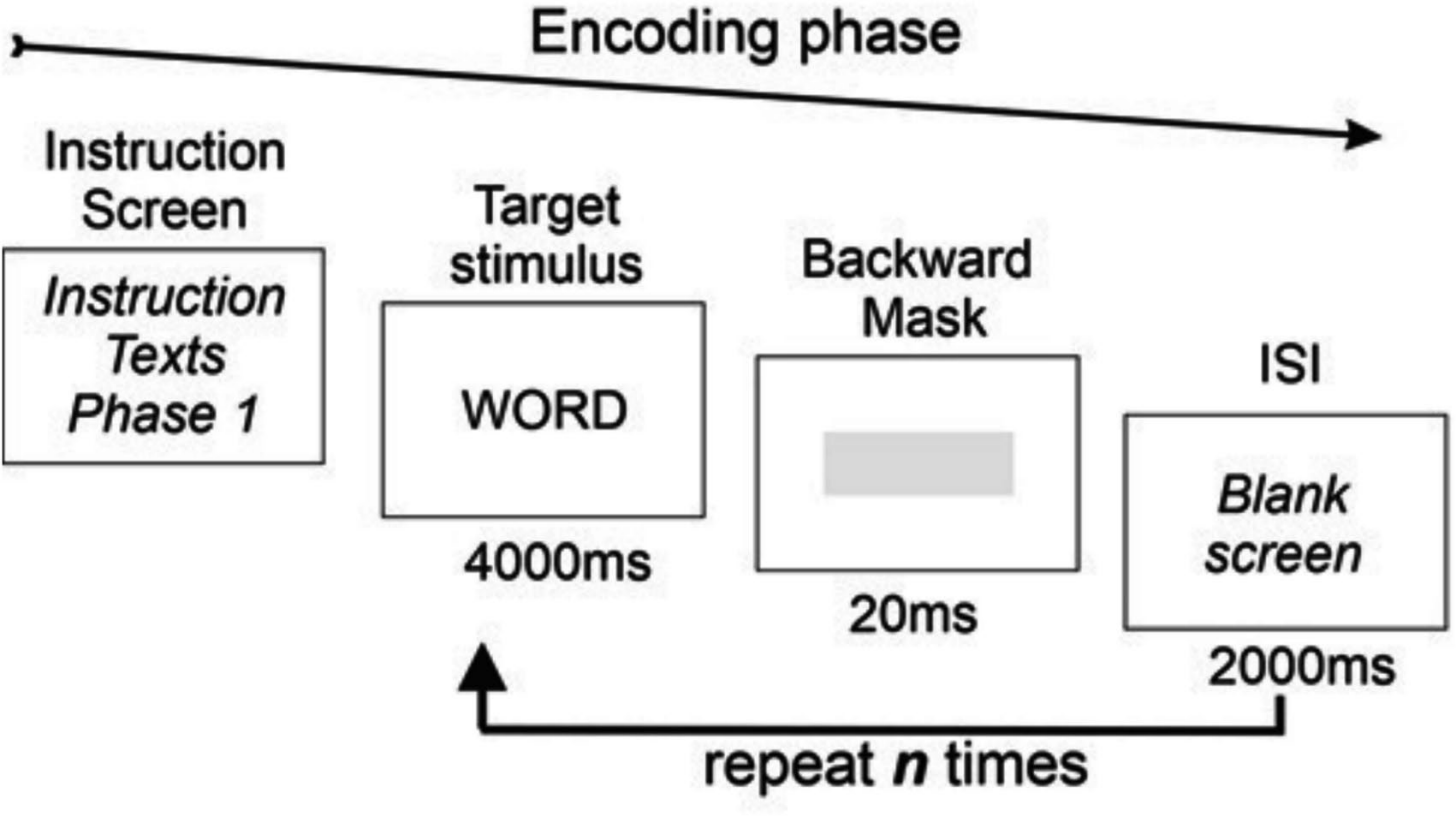
Diagram of the stimulus presentation during the encoding phase.

During the encoding task, stimulus word exposure time was 4,000 ms with an interstimulus interval ISI of 2000 ms (see Figure 1).

### 2.3 EEG recordings

EEG was recorded with a sample rate of 512 Hz and cut-off frequencies of 0.1–50 Hz via 19 Ag/AgCl cup electrodes fixed to the scalp with electrolyte gel at electrode positions with impedances < 5 kOhm, which included the 19 standard electrodes of the 10–20 system (Penfield and Jasper, 1954), Cz as recording reference. Schwarzer EEG-29 T recording system with Coherence version 6.1.3.417 application software (Natus Europe GmbH) was used.

### 2.4 EEG data preprocessing and data processing

EEG data were analyzed offline using Matlab (The MathWorks, Inc.) based EEG analysis software products, EEGLAB and ERPLAB,^2^ with some custom processing scripts.

Data prepossessing and further EEG analyses were performed with EEGLAB. Cz recorded reference electrode has been re-referencing to average reference, 50 Hz line noise in EEG signals was rejected using bandpass filters with a value of 50 Hz after that cut-off frequency of 0.1–50 Hz was applied. Then, EEG signals with performance errors or remaining artifacts exceeding ± 120 μV in any channel and eye-blinked artifacts were rejected using the ICA procedure (EEGLAB Wiki, 2024) from data before processing.

The study included the aforementioned set of ROI, representing memory encoding and verbal information processing: F3, F7, C3, P3, T3, and T5. The event-related potential (ERP) and time-frequency analysis (TF) for event-related spectral perturbation (ERSP) were calculated for −200 to 800 ms for each stimulus presentation using the ERPLAB Study pipeline for each group of stimuli during the encoding phase. TF was calculated with the following parameters: cycles 3 0.8, nfreqs of 100, and ntimesout of 200.

### 2.5 Statistical analysis

The ANOVA with a list length as a within-subjects factor was calculated for the top-level analysis of the TF analysis, followed by the independent unequal variance two-tailed t-test for ERP and TF. The calculations were performed within the EEGLAB using the STUDY (EEGLAB Wiki, 2024) function with EEGLAB statistics, the statistical threshold level “exact,” and permutation statistics for the parametric model, ensuring the robustness of inferences. They compared ERP and TF in different lists lengths within the windows of 0 to 800 ms for ERP and −100 to 700 ms for TF. For all tests, the p-level <0.05 was considered significant.

## 3 Results

### 3.1 ERP analysis

**Figure 2:**
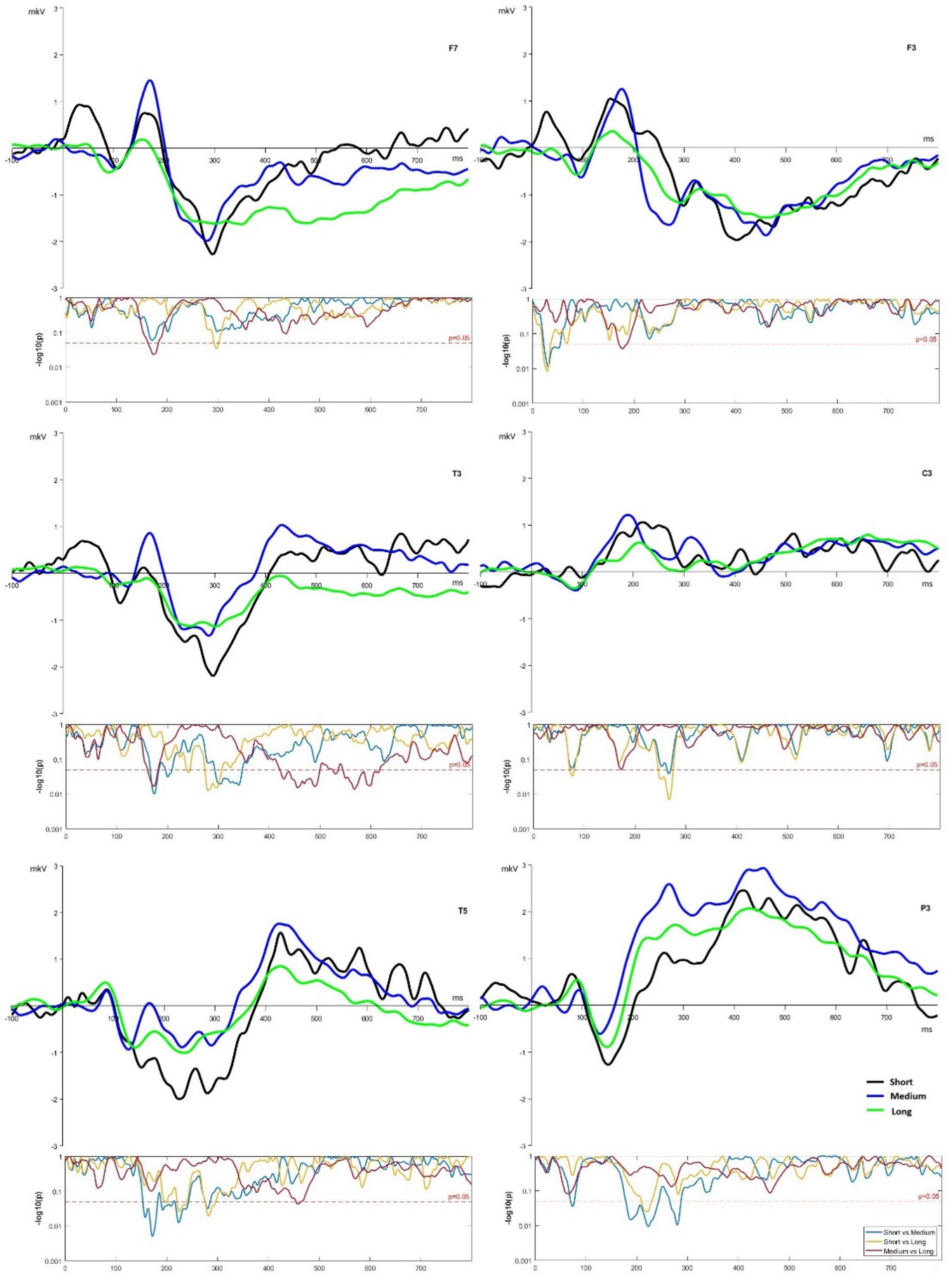
ERP of F3, F7, C3, T3, T5, and P3 channels for short, medium, and long word lists.

The main differences between all three conditions (short, medium, and long word list lengths) were observed in the temporal and parietal areas of the brain under the P3, T3, and T5 electrodes (see Figure 2).

For ROI F7, amplitude differences were observed at the time slot 140–200 ms with significantly different medium vs. long lists at *p* < 0.05, and the long lists curve then had some differences in the period after 350 ms (see Figure 2) for the long list, it indicates less intensive but longer encoding. In contrast, there were no differences within the same window for ROI F3. There were main significant differences for short lists in the time slot at 30–70 ms from others lists, and long lists had significant differences from medium lists at around 180 ms.

ROI C3 demonstrated lower activation under the long list condition within time slots 140–340 ms from other list lengths, including significant differences between long vs. short lists in the 250–290 ms window. For ROI T3 and T5, the pattern with a significant difference for short vs. medium lists was observed around the classical N2 spike. In both ROI electrodes, the short list amplitude was lower at 200–400 ms time slot. The medium list demonstrated the highest spike in T3 around 400 ms (see Figure 2) and significantly differed from the long list within the window of 420–620 ms. In ROI P3, the short list condition demonstrated the most pronounced spike at around 150 ms, and the medium list had a higher amplitude spike after 200 ms (see Figure 2). The medium list showed the most significant differences from the short one at 170–290 ms. Although significant results were observed in all ROI electrodes (see Figure 2), ROI P3, T3, and T5 demonstrated highly significant differences within any temporal windows under investigation. These ROIs can indicate a highly variable load on Wernicke’s area (Tremblay and Dick, 2016), which is responsible for language comprehension and the meaning of words. Overall, long lists had smaller amplitudes, especially at more lateralized ROI channels, and at F7, T3, and T5, they showed nonsignificant differences at a later time slot, after 400 ms.

**Figure 3:**
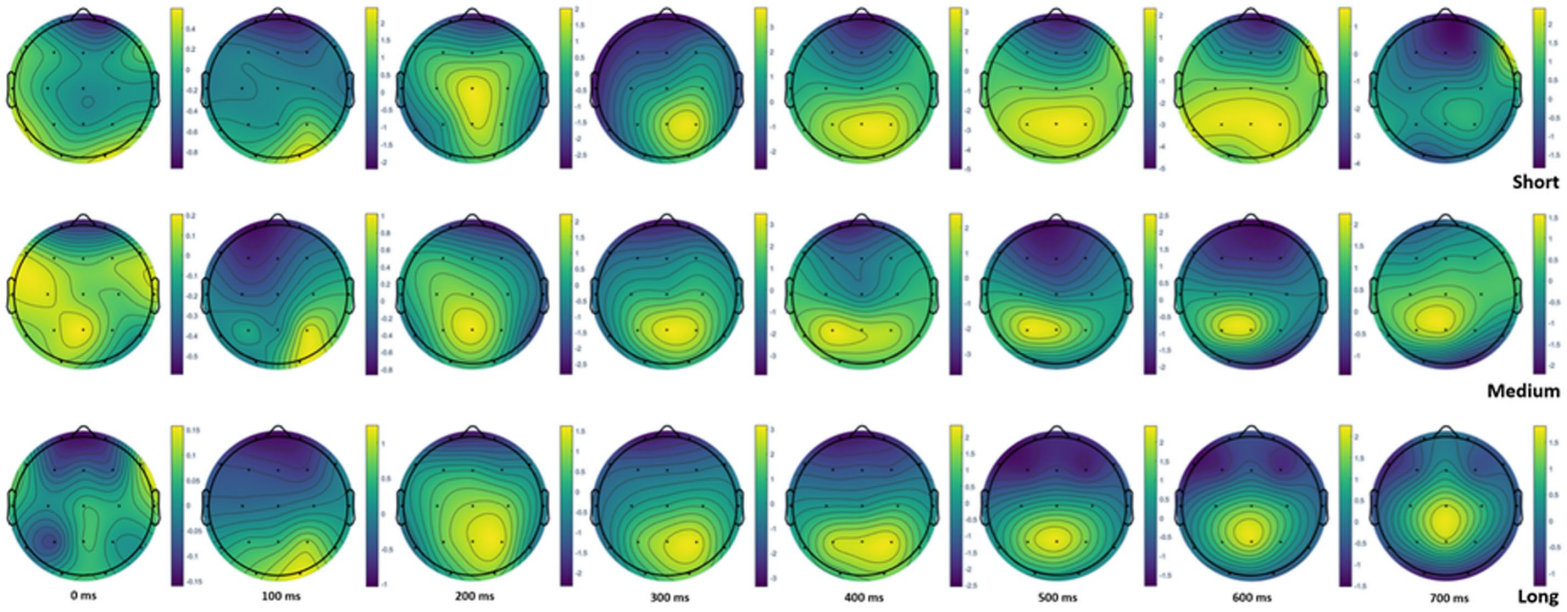
Head topoplot at 0, 100, 200, 300, 400, 500, 600, and 700 ms for short, medium, and long word lists.

Additional head topoplot visualization (Figure 3) demonstrated that information encoding at 400–600 ms time windows had different patterns for short versus medium and long lists. Similarities in the observed patterns and consequent differences in ERP under the short list condition may suggest that, in the medium list length interval (30–59 items), the brain modifies the mode of information processing and maintains it in the long list (up to 160 items).

### 3.2 TF analysis

**Figure 4:**
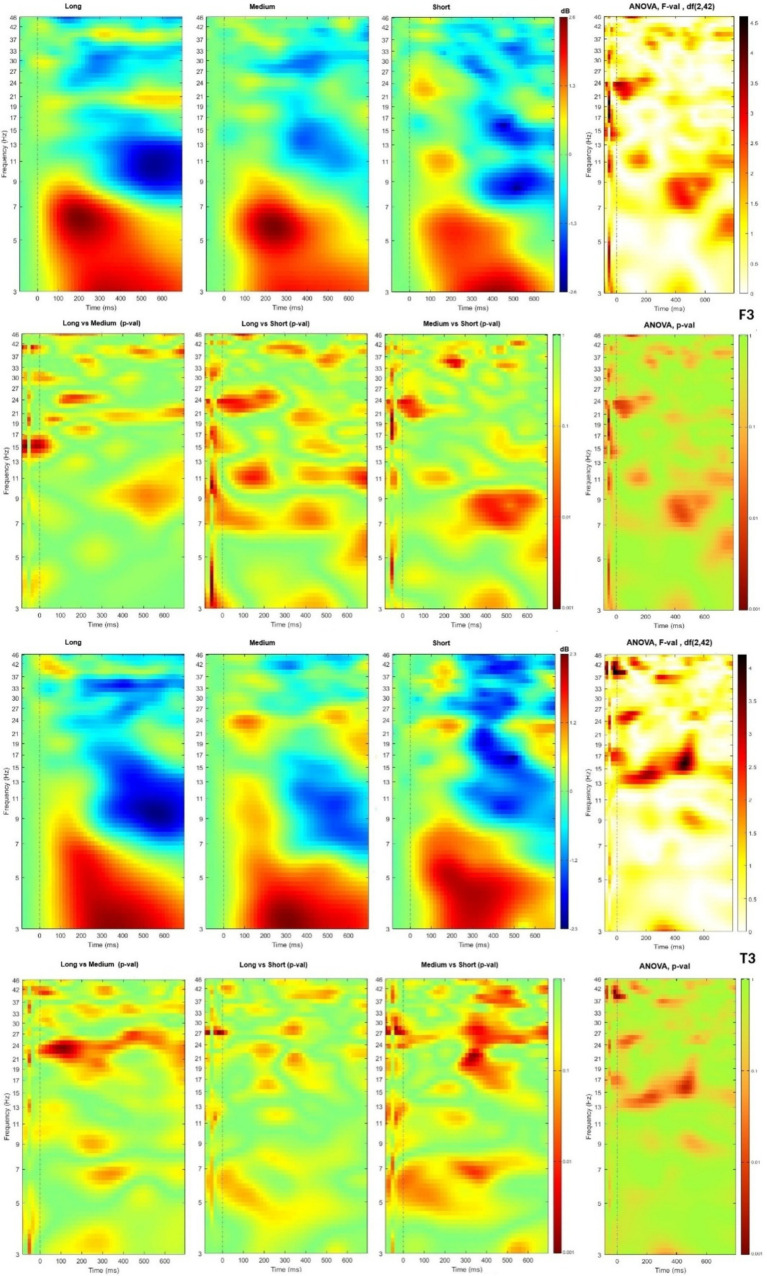
Time-frequency plot of different lists length in channels F3 and T3 with t-tests and ANOVA *F*-value and *p*-value matrixes.

Data were presented from two ROI under F7 and T3 electrodes, corresponding to Broka and Wernicke areas (see Figure 4). In both channels, two activity regions have presented high spectral power at theta band at 100 to 500 ms and low spectral power at the alpha band at time windows from 300 ms. In the T3, beta bands occurred at 300–400 ms time slot, together with alpha bands.

ANOVA showed significant differences among list lengths at lower-beta (13–19 Hz) at 100–600 ms time slot in T3 and higher-beta (19–30 Hz) at about 400 ms in F3. Theta wave bands have been observed in the F3 channel at time slot till 450 ms, and the alpha wave bands in T3 at 440–600 ms time slot.

In t-tests, the short length lists differed in F3 from other lists at higher beta (21–25 Hz) at the time slot around 100 ms. Another significant difference was between the short and medium lists in the alpha band. It was observed after 300 ms, but in short vs. long lists - during all observation time. Lower-gamma significantly differed in time slot 200–500 ms. Long and medium lists also had significant differences in the alpha waves band at 400–600 ms time slot, in beta during all observation time, and in gamma after 200 ms. T3 demonstrated the main differences between short and medium lists with a significant difference in theta band till 500 ms and higher-beta and lower-gamma after 270 ms with the most pronounced differences at the 300–400 ms time slot. The same but less expressed patterns were observed for short vs. long lists. In comparison with F3, T3 demonstrated that the beta band had more pronounced differences, and theta bands were activated during 200–400 ms for two longer lists.

## 4 Discussion

Observed differences in the window after 450 ms in ROI F3 and F7 (see Figure 2) point to the more active involvement of a more lateralized (F7) left-hemispheric regions during encoding under different conditions (Wiesman et al., 2021). It also points to the intensive involvement of working memory regions in processing the most extended sets of stimuli (Jeneson and Squire, 2012). A similar tendency of lateralization was observed in ROI T3 vs. ROI C3. ROI C3 is associated with a motor component or behavior and reactions to stimuli. At the same time, T3 activity indicates temporal lobe involvement in processing lists of lengths, exceeding the volume of working memory (Jeneson and Squire, 2012). Simultaneously, a low activation level under the highest list length in ROI T3 and T5 points to the possible exhaustion of neural resources for encoding under increasing cognitive load (Wiesman et al., 2021). A previous study (Senkfor and Van Petten, 1998) found that correctly recognizing old words elicited more positive ERPs than new words, beginning from 400 ms. Therefore, a low activation level under the highest list length may relate to a worse possibility of recognizing verbal information, leading to lower activation of these brain regions.

The same pattern of the longer lists shows the absence of the effect of lateralization in ROI P3 (less lateralized) and T5 (more lateralized) can be associated with continuous involvement of Wernicke’s area in verbal information processing (Tremblay and Dick, 2016). P3 is one of the most reported ERP parameters during short-term memory retrieval. Ergen et al. (2012) suggest that the right-lateralized positivity in the pre-P3 period reflects the memory scanning process, followed by the P3 peak with midline parietal topography reflecting the decision-making process. Simultaneously, the involvement of parietal and temporal regions in encoding (Jeneson and Squire, 2012; Chai et al., 2018) and verbal information processing (Fedorenko and Blank, 2020; Tremblay and Dick, 2016) vary in a nonlinear mode. There was no simple increase or decrease in ERP characteristics under observed experimental conditions. These tendencies indicate a need for a more detailed investigation of the relationship between the length of the word list and brain functioning.

Exploration of brain activation within the topoplot indicated more contrasted anteroposterior activation of brain regions in the windows of 400–600 ms for medium and long lists than for short lists. This trend is in line with the greater involvement of the parietal zone in information processing during encoding (Jeneson and Squire, 2012). Within the interval of 30–59 units, it is possible to observe a turning point for involving brain resources in the encoding. Literature data show that event-related potentials with positivity around 450 ms and negativity around 500 ms and delta and theta event-related oscillations correlate with memory performance (Erdogdu et al., 2019). However, there is a need for further quantification of this tendency in the frame of integrated assessment of brain activity.

Our findings on brain oscillations confirm that theta band plays a significant role during the encoding of information (Osipova et al., 2006; Gruber et al., 2013). We observed theta band differences under T3 ROI in the case of short and medium word lists where the theta band significantly differs till the time of 500 ms. Unfortunately, gamma activity was represented minimally (lower gamma) because of equipment limitations and filtering out most of the band.

The results on TF with alpha band differences, especially in short vs. medium lists, correspond with the observed encoding-related alpha/beta power associations with episodic memory (Griffiths et al., 2021). Literature data suggest that increased total alpha activity was found in the retention interval for the memory as compared to the perception condition (Herrmann et al., 2004). Klimesch et al. (2005) suggest that the theta band reflects working memory functions, whereas upper alpha may be important for the reactivation of long-term memory codes in short-term memory.

A small sample of respondents and large intervals in splitting word lists into conditions formed the study’s main limitations. Not all a large number of participants disallow us from calculating the ERP in short lists 1–8, which correspond with short time memory, because of ERP methodological limitations regarding the number of observations. The time-frequency method could be a better way to process data regarding this interval. Simultaneously, long lists can also be explored in greater detail, considering the limitations of neural structures (Hill and Dunbar, 2003; Whitehead, 2010). The sample size and homogeneity regarding gender limit the generalization of explored effects and require the involvement of larger and gender-balanced samples in future studies. In addition, focusing on the left-hemispheric activity can be changed in further studies to reflect total brain activity. Better spatiotemporal and brain network-centered activity tracking (Tremblay and Dick, 2016) over classical language centers will open new, more precise perspectives for languages and memory studies.

In summary, our exploratory study confirms an association between left-hemispheric neural activity and the list length during the encoding task. Language processing and comprehension regions significantly differed in activation under different list lengths, pointing to a possible nonlinear trend in length-associated effects. More lateralized regions in the frontal and temporal zones may demonstrate more pronounced differences in the encoding process under different encoding loads than less lateralized regions. The observed synchronization of the theta wave power seems to play a role in encoding and processing verbal information over the brain regions involved in this task.

## Data Availability

The original contributions presented in the study are included in the article/supplementary material, further inquiries can be directed to the corresponding author.

## Footnotes

^1^ http://psychtoolbox.org/

^2^ http://sccn.ucsd.edu
